# A Review of Irradiation Damage and Effects in α-Uranium

**DOI:** 10.3390/ma15124106

**Published:** 2022-06-09

**Authors:** Arunkumar Seshadri, Andrea M. Jokisaari, Cheng Sun

**Affiliations:** Idaho National Laboratory, Idaho Falls, ID 83415, USA; arunkumar.seshadri@inl.gov (A.S.); andrea.jokisaari@inl.gov (A.M.J.)

**Keywords:** α-uranium, irradiation damage, irradiation effects, mechanical properties, physical properties

## Abstract

Understanding irradiation damage and effects in α-uranium (α-U) is critical to modeling the behavior of U-based metallic fuels. The aim of this review is to address the renewed interest in U-based metallic fuels by examining the state-of-the-art knowledge associated with the effect of irradiation on the microstructure, dimensional changes, and properties of α-U. We critically review the research progress on irradiation-induced growth and swelling, the enhancement of plastic flow and superplasticity by irradiation, and the effect of irradiation on thermal and electrical properties of α-U. Finally, we outline the research directions that require advancements, specifically the need to carry out fundamental research on several of the less understood mechanisms of irradiation damage and effects in α-U.

## 1. Introduction

Although the current light water reactors predominantly use uranium dioxide as the major fuel elements, metallic uranium and uranium alloys were extensively considered for fast reactor applications in 1950s and 60s due to their higher fissile and fertile material density compared to oxides. Recently, the nuclear engineering community has renewed interest in several metallic uranium concepts for use in Generation IV reactor designs [1,2]. Under the Department of Energy, the advanced fuels campaign is focusing on metallic uranium concepts for sodium fast reactors [3]. α-U is an important allotrope of solid uranium observed at low temperatures (<940 K). Much of the research on α-uranium occurred after World War II to solve the persistent problems encountered in preparing uranium metal for a nuclear fuel and for plutonium production (particularly at Hanford and Savannah River sites). There was a large effort in the late 1940s and early 1950s, particularly at Argonne National laboratory and Knolls Atomic Power Laboratory, to develop an understanding of the anisotropic thermal expansion, irradiation-induced swelling and irradiation creep of α-uranium. Some of the comprehensive reviews on the properties of metallic uranium with a focus on irradiation effects were reported in late 1950s and 60s [4].

α-U exhibits several unusual properties, including high-pressure superconductivity, high plastic flow induced by irradiation growth, and massive, catastrophic irradiation swelling (referred to as “breakaway swelling”) at a temperature of around 923 K. Several of these properties are not completely understood even today. However, the fundamental research on irradiation-induced dimensional growth of α-U progressed into the late 1960s and early 1970s, which also included a worldwide effort in measuring its physical properties (such as electrical resistivity and thermal conductivity) and mechanical properties (such as plastic deformation). The nuclear industry, however, gradually moved forward with ceramic UO_2_ fuels, leading to a rapid decline in metallic uranium research. However, the condensed matter physics community had continued interest in α-U as a superconductor. In 1994, Lander et al. [5] comprehensively covered the properties of unirradiated uranium, primarily focusing on its electronic and mechanical properties. However, α-U has witnessed renewed interest [3] in the nuclear industry in the last decade, primarily because it is a component of certain metallic fuels for fast reactors. 

The present review focuses on the fundamental insights available from the research over the last few decades, with focus on the recent research progress on irradiation damage and effects in α-U. This review starts by tabulating the crystal structure and mechanical and physical properties of α-U in Section 2. In Section 3, irradiation-induced microstructural and dimensional changes in α-U are reviewed. Following this, we summarize the effect of irradiation on its mechanical and physical properties. Finally, in Section 4, we provide a brief outlook for future research.

## 2. Properties of α-U

### 2.1. Crystallography and Fundamental Defect Kinetics

**Figure 1:**
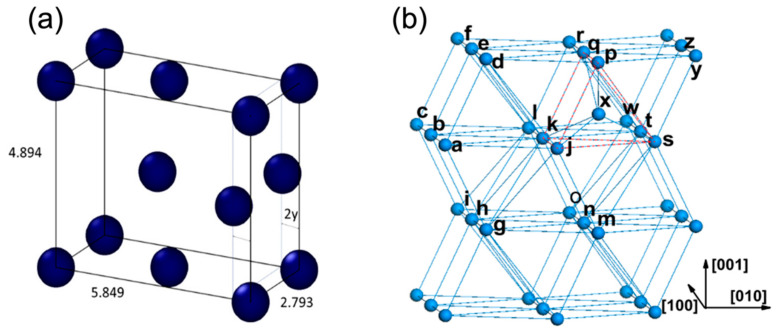
(**a**) α-U unit cell with lattice parameters estimated by Beeler (reprinted and adapted with permission from [7], 2022, Elsevier). (**b**) schematic structure of α-U and the most stable self-interstitial configuration (reprinted and adapted with permission from [8], 2022, IOP publishing, Ltd).

The α phase of uranium consists of corrugated sheets of atoms in a highly asymmetrical face-centered orthorhombic structure [6]. Beeler et al. calculated the lattice constants of α-U with a = 2.793 Å, b = 5.849 Å, c = 4.894 Å, as seen in Figure 1a [7]. The structure of α-U is made up of close-packed corrugated planes of atoms, including planes parallel to the densely packed flat (010) plane and planes parallel to the [100] axis. The interatomic distances of corrugated planes (e.g., line segments ab = 2.70 and ad = 2.80 Å in Figure 1b) are smaller than interatomic distances between the corrugated planes (e.g., line segment ak = 3.25 and dk = 3.34 Å in Figure 1b) [8]. This corrugated structure is responsible for the anisotropy of physical properties of α-U. There is only one type of lattice site, tetrahedral interstitial site, pentahedron site, and single vacancy site. However, there exist several types of self-interstitial configurations (eight tetrahedral, eight pentahedral pyramidal and four octahedral interstitial sites). The most stable configuration is schematically shown in Figure 1b, where atom X is in the interstice of a pentahedron.

**Figure 2:**
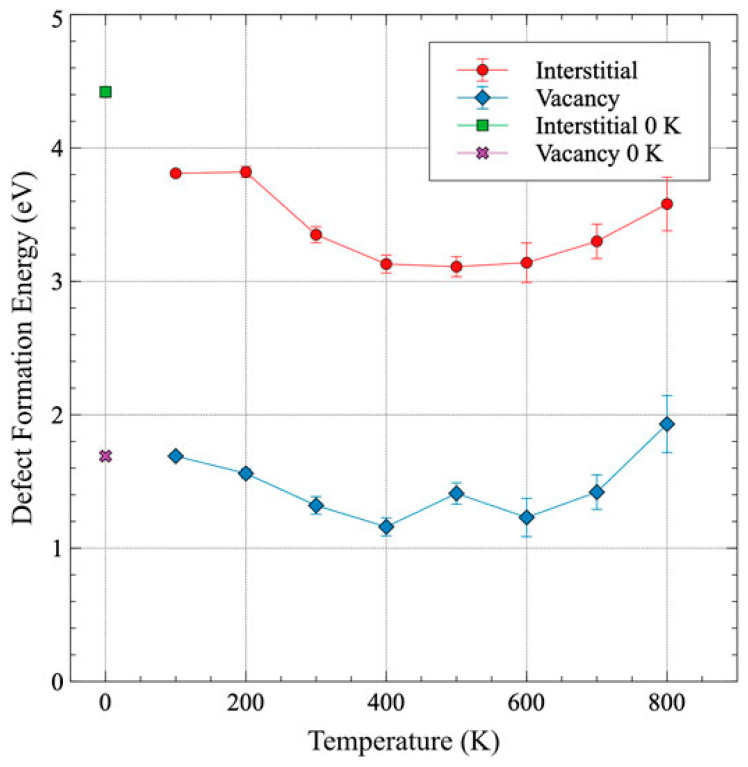
Formation energy of interstitial and vacancy as a function of temperature (reprinted and adapted with permission from [10].

Recent calculations of point defect formation and displacement energies indicate that radiation damage is expected to be less significant in the α-phase compared to the γ-phase. The threshold displacement energy of α-U was estimated using density-functional theory (DFT) and molecular dynamics (MD) calculations [8,9]. The threshold displacement energy of α-U was estimated to be 63.4 eV at 800 K. Compared to 35.6 eV in γ-U at 800 K, more energy is required to displace an atom from a lattice site in α-U and the number of collision cascades from the same primary knock-on energy is reduced. The minimum threshold displacement energy (~20 eV) exists along the [100] to [010] directions and hence these are expected be directions of easier defect creation. Beeler et al. [9] also reported the defect formation energies and diffusion coefficients in α-U at 600 K and 800 K through ab initio MD simulations. The estimated interstitial and vacancy diffusion coefficient in the 600–800 K range was 2.172 × 10^−7^ and 1.148 × 10^−7^ m^2^/s, respectively. More recently, in [10] the variation in defect formation energy with temperature was studied via ab initio MD and is shown in Figure 2. The defect formation energy for both vacancy and interstitial defects tends to slightly decrease until 400 K and then increases with increasing temperature.

### 2.2. Electronic Properties

The electronic structure of uranium is dominated by 5f electron states that form narrow bands that tend to bond the atoms in complex and distorted ways. DFT results reveal that 5f orbitals are active in the chemical bonding for uranium [11]. The behavior of 5f electrons of uranium is observed to be similar to that of the d orbital electrons in the transition elements [12]. Compared with the bulk atoms, the deficit of atoms near the uranium surface breaks the balance between the delocalization and localization of 5f electrons, leading to the electrons changing from delocalized to localized. As the electrons change from delocalized to localized, the arrangement of the surface atoms is interrupted and the total energy of the surface increases. This principally leads to a high surface energy, in turn leading to a high affinity for hydrogen, oxygen and carbon [11,13]. 

The research on the electronic properties of α-U was mainly motivated by the anomalous electronic configuration of α-U [5] and the discovery of superconductivity in α-U [14]. A long-standing debate on the description of the 5f elements involved whether they should be called actinides, in which the 5f shell was progressively filled, or thorides, in which the 6d shell progressively filled. From the chemical point of view, this question was effectively settled by the production of the heavier elements Np and Pu and the early study of their spectroscopic properties, revealing the actinide nature of α-U. Superconductivity was discovered in α-U in 1942 by Aschermann and Justin and was first reported by Mott [14]. However, the existence of intrinsic superconductivity in α-U (i.e., superconductivity of α-U in the pure undoped state) is a topic of discussion even today. Multiple investigations in the 1950s found a range of superconductivity transition temperatures [5] depending on the purity of the samples. Conversely, several late 1960s studies indicated the possibility of superconductivity being the result of grain-boundary filamentary sheathes rather than bulk superconductivity, indicating that superconductivity is not intrinsic to α-U [15]. In fact, Geballe et al. [16] suggested that the variations in superconductive properties were correlated to the intergranular strains resulting from anisotropic expansion in α-U. 

Three phase transitions occur in α-U below 43 K due to the presence of charge density waves and were postulated to affect the intrinsic superconductivity of α-U [17]. Charge density waves (CDW) occur when there is a spatial modulation of the electron density [18]. This CDW is typically associated with a mass density wave of the positively charged ions which make up the crystal lattice. The possibility of CDW formation is enhanced in lower-dimensional systems (1D and 2D materials) because the simple structures involved lead to a high probability of favorable Fermi surface nesting (i.e., the shifting and coincidence of two Fermi surfaces that are required for CDW formation). In bulk materials, CDW phenomena are rather rare because of the unlikelihood of Fermi surface nesting. Uranium is the only element in the periodic table to exhibit a phase transition to CDW states at ambient pressure. The three phase transitions can be seen as a drastic change in the derivative of the electric resistivity vs. temperature [18]. 

Interestingly, these phase changes do not lead to any changes to the orthorhombic structure, but primarily the changes are observed on the thermal expansion coefficient, the heat capacity and the electronic charge density. Studies on different materials have indicated that CDWs compete with superconductivity for conduction electrons, and this competition is hypothesized to be the reason for the absence of intrinsic superconductivity in α-U. Attempts were made to understand the intrinsic superconductivity in α-U in the absence of such phase transitions by increasing the external pressure, which was expected to suppress the phase transition. The increased critical temperature in single crystal α-U with the application of pressure was precisely documented in [19], confirming intrinsic superconductivity in α-U in the absence of CDW-caused phase transitions. However, some recent studies [20] do strongly favor the presence of superconductivity in α-U at ambient pressure. 

The average electrical resistivity of α-U at room temperature is around 0.2 µΩm [21] which is similar to iron. Few researchers have measured the increased electrical resistivity of α-U with respect to temperature [21,22]. The electrical resistivity of α-U is also anisotropic, with the [100] direction having the highest resistivity and [010] the lowest resistivity at elevated temperatures, although at low temperatures (<400 K), [100] exhibits slightly higher resistivity compared to [001]. 

### 2.3. Mechanical Properties 

**Figure 3:**
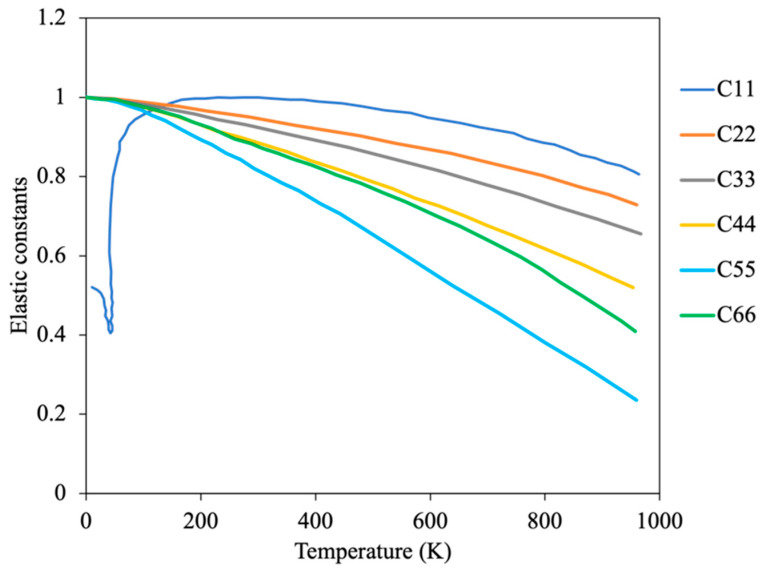
Temperature dependent elastic constants of α-U (Reprinted/adapted with permission from Ref. [23], 2022, Elsevier).

The anisotropic nature of the mechanical properties in α-U was the major motivation behind several early experimental and recent modeling efforts. The elastic constant variation with temperature in single crystals was experimentally determined by Fischer [23] and is shown in Figure 3. All the elastic constants exhibit increasing negative curvature with temperature above room temperature, such that significant softening occurs as the beta phase transition temperature is approached. However, there is a second order transition in C11 at the low temperature CDW-induced phase change around 43 K [23]. The moduli decrease monotonically with temperature above 273 K, reflecting the changes in the single-crystal constants [23]. In addition, the polycrystalline mechanical properties were computed using single crystal elastic constants in [7] by applying the Voigt–Reuss–Hill approximation. The Young’s modulus in the [010] direction (normal to the corrugated planes) is roughly 30% less than in [100] or [001], indicating the relatively weaker bonding between corrugated planes versus within the planes. The Poisson’s ratio of α-U was also observed to be anisotropic and is not a strong function of temperature below 800 K. 

**Figure 4:**
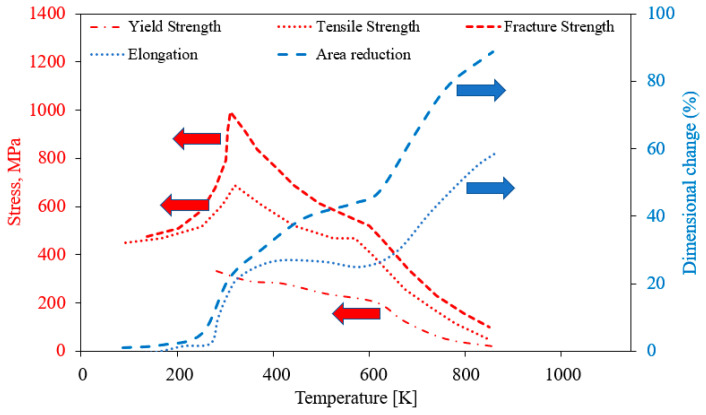
Mechanical properties of α-U as a function of temperature (Reprinted/adapted with permission from Ref. [24], 2022, Springer Nature). Red arrow represents Stress (MPa), and blue Dimensional change (%).

The mechanical properties of polycrystalline α-U vary substantially with temperature, as shown in Figure 4. The tightly bonded corrugated basal planes in α-U result in a very limited number of slip systems, such that room-temperature slip occurs only on these corrugated basal planes parallel to the direction of the corrugations ((010) [100] system) [24]. This singular slip system is observed to support macroscopic ductility at room temperature only with the activation of twinning. Above approximately 525 K, additional slip systems such as (001) [100] and (110) [l10] slip systems become active, and the material becomes softer and much more ductile. At temperatures below 273 K, slip becomes more difficult, even on the preferred (010) [100] system. A transition from ductile to brittle behavior accompanies this decrease in slip and increase in twinning.

### 2.4. Thermal Properties

**Figure 5:**
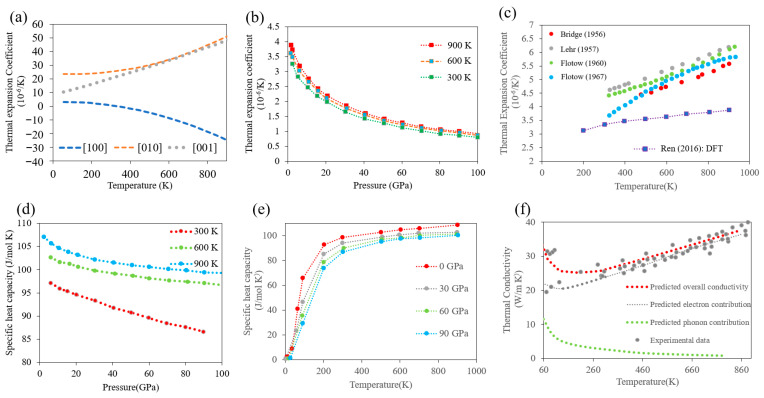
Thermal properties of α-U: (**a**) anisotropic thermal expansion coefficient in single crystals with different orientation (Reprinted/adapted from Ref. [19], 2022, Elsevier), (**b**) thermal expansion coefficient as a function of pressure ((“Reprinted/adapted from Ref. [28], 2022, Elsevier), (**c**) thermal expansion coefficient as a function of temperature: comparisons between the DFT studies [28] and experiments [26,29,30,31] (“Reprinted/adapted from Ref. [28], 2022, Elsevier), (**d**) specific heat capacity as a function of pressure (“Reprinted/adapted from Ref. [28], 2022,Elsevier)), (**e**) specific heat capacity as a function of temperature (“Reprinted/adapted from Ref. [28], 2022, Elsevier), (**f**) thermal conductivity (DFT studies adapted from [20]) compared with available experimental results (Adapted from Ref. [20]).

Electronic charge carriers play a dominant role in the thermal properties of α-U, such as thermal conductivity. The literature suggests that uranium follows the Wiedmann–Franz law [25] for the proportionality between electronic conductivity and thermal conductivity, which states that the ratio of the electronic contribution of the thermal conductivity to the electrical conductivity of a metal is proportional only to the temperature. The first-principles calculations of Hin [22] show that temperature-dependent thermal conductivity of both polycrystalline and single crystal α-U follow similar trends as the electrical resistivity. Both the thermal conductivity and the electrical resistivity of single crystal α-U exhibit anisotropic behavior [17]. The average thermal conductivity of α-U at room temperature is about 25 W/mK, which is approximately an order of magnitude lower than copper. Single crystal α-U exhibits anisotropic thermal conductivity, and the conductivity along the [001] direction (normal to the corrugated plane) is about 25% lower than along the [010] direction [17]. A comparison of DFT and experimental results on thermal conductivity are plotted in Figure 5f. A near linear increase in thermal conductivity from 300–900 K is observed, primarily because of the dominant electronic contribution to the thermal conductivity. At low temperatures, the influence of defects (e.g., point defects, impurities, and dislocations) can significantly impact the thermal conductivity of α-U, and hence the observed discrepancies between experimental results at low temperature may be the result of differences in the sample chemistry and crystal defects [22].

The thermal expansion coefficient values of α-U are anisotropic, with different, positive coefficients along the [100] and [001] axes, but the values along the [010] axis are negative at all temperatures. It should be noted that although many non-cubic crystal metals exhibit anisotropic thermal expansion, α-U is extremely unusual in having a negative thermal expansion coefficient. The first X-ray diffraction data for the anisotropy of the thermal expansion above room temperature were reported by Bridge et al. in 1956 [26] for polycrystalline α-U. However, more reliable data from dilatometer measurements were provided by Lloyd [27] for single crystals with different orientations (shown in Figure 5a). In addition, the thermal expansion coefficient of a polycrystalline α-U is a strong function of pressure, but only varies significantly with temperature at low temperatures (<150 K) (Figure 5b,c). Some discrepancies in the measured thermal expansion may be due to the different impurity levels of the samples.

## 3. Irradiation Damage and Effects in α-U

### 3.1. Irradiation-Induced Growth and Swelling

Irradiated polycrystalline α-U was initially characterized macroscopically in the late 1940s and early 1950s, and samples were observed to grow in length and/or increase in volume (swell) at a rapid rate when irradiated. Understanding the dimensional instability arising from radiation is important for reactor component design. The early results considered the sample deformation to be a function of material fabrication, working temperature and thermal history. Some studies [32,33] revealed that the swelling rate of α-U could be reduced with small additions of low solubility alloying elements, especially silicon, aluminum or carbon. It was also observed that thermal cycling produces damage in unirradiated uranium roughly like the radiation effects in appearance. Pugh [34] theorized that the mechanism behind irradiation-induced elongation was the same as thermal cycling. Though it is now known that there is apparently no relationship, at that stage it was hypothesized that thermal cycling could be a stand-in for irradiation. In polycrystalline α-U, enormous irradiation-induced elongation was observed especially at high burnup. Note that the macroscopic growth in polycrystalline samples is not the same as the volume-conservative growth in single crystals, though the phenomena are related. We are careful to specify polycrystalline or single crystalline sample states in this discussion. The first detailed analysis of polycrystalline sample growth was presented by Kittel and Paine [35] in 1958, on the irradiation growth of uranium that had been processed with different conditions. The irradiation was conducted at the Material Test Reactor (MTR) with irradiation temperatures up to 493 K and burnups up to 1.82 at% (no details were provided on sample purity). This study indicated that irradiation growth is a strong function of burnup. Around the same time, Paine and Kittel [36] measured the volume-conservative irradiation growth rate with respect to burnup for single crystal α-U specimens, though the irradiation temperature is not well known. This work determined that single crystal specimens grow in the [010] direction, shrink in the [100] direction, and remain the same in the [001] direction, and that the overall volume of the crystal is conserved during irradiation growth. The discovery of irradiation growth in single crystal uranium immediately leads to a hypothesis for the multiple observed irradiation-induced deformation behaviors in polycrystalline material and the variation in behavior depending on the sample processing. The collective deformation behavior of multiple individual grains behaving anisotropically should be influenced by grain size and grain texture.

Initial studies motivated by macroscopic engineering concerns were succeeded by more scientific endeavors to understand the physics of the irradiation growth behavior. During 1952–1962, three different mechanisms were postulated. The first mechanism, proposed by Pugh [34], combined the insights from Cahn [37] on the observed twin and slip systems and the idea of local thermal expansion induced by fission spikes to propose that irradiation growth resulted from irreversible plastic deformation in the heated region surrounding a fission track. Interestingly, Pugh’s theoretical predictions on anisotropic dimensional change were experimentally observed in most of the single crystal swelling studies [38]. The second mechanism, proposed by Seigle and Opinsky [39], hypothesized that the lattice imperfections generated in α-U during irradiation could anisostropically diffuse to and be eliminated at grain boundaries and free surfaces. In this case, the interstitial atoms would add to the lattice, producing an extension, and vacancies would subtract from the lattice, producing a contraction of the grain at its boundaries. If sufficient diffusion occurs and the flux remains unbalanced, macroscopic growth could be observed. Using simplified considerations of the interstitial positions in the α-U lattice and the possible diffusion routes, they theorized that the maximum interstitial migration would be along the [010] direction and the maximum vacancy migration would be along the [100] direction, implying lattice growth along [010] and lattice shrinkage along [100]. This is qualitatively the same as the observed single crystal deformation, but the actual anisotropic diffusion behavior of interstitials and vacancies separately and their interacting behavior in a crystal remains to be determined to confirm or reject this mechanism. The third mechanism, proposed by Buckley [40], hypothesized that the irradiation-induced elongation and contraction of uranium crystals are the result of the anisotropic condensation of vacancy and interstitial defects in planar clusters within the volume associated with a U-235 fission event. Based on this hypothesis, planar clusters of interstitial atoms increase the dimension of a crystal in the [010] direction by creating extra (010) planes, and planar clusters of vacancies erode existing (100) planes, causing contraction in the [100] direction. Around the same time, Hudson et al. [41] performed the first thin film transmission electron microscopic measurements and observed the formation of dislocation loops in α-U after neutron irradiation.

**Figure 6:**
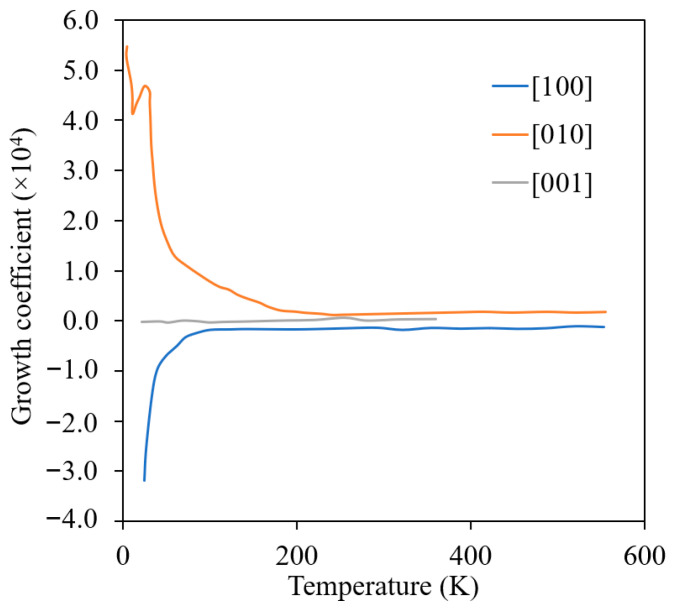
Effect of irradiation temperature on the growth coefficient of α-U single crystals (Reprinted/adapted with permission from Ref. [42], 2022, Taylor & Francis).

To date, the details of irradiation growth in α-U remain unclear, including the exact mechanism or mechanisms of irradiation growth in single crystals, the single-crystal irradiation growth behavior at elevated temperatures, and the impact of interphase boundaries, grain boundaries and solid solution impurities. Loomis and Gerber [42] carried out a detailed investigation and studied the irradiation-induced dimensional change in single crystals and lineage crystals (crystals with low-angle grain boundaries) over a wide range of temperature (shown in Figure 6). Note that in Figure 6, the growth coefficient is defined as the ratio of change in length to the initial length per fraction of U fission atoms. They also reported the markedly enhanced dimensional changes in the [010] and [100] directions below the irradiation temperature of 50 K. The enhanced dimensional change was hypothesized to be the result of increased point-defect mobility due to the CDW-state observed below 43 K. However, it remains unclear whether irradiation-induced crystal growth completely stops at high temperature. Hudson observed that the presumed dislocation loops were randomly dispersed at low temperature but showed a very marked coplanar distribution at high temperatures (>623 K). The advanced characterization of α-zirconium, which also exhibits anisotropic growth, has suggested that defect clustering and interstitial and vacancy loop formation is most likely in that material [43]. They proposed this is to be a sequential result of the mechanism of movement into rows by glide, as the ease of deformation by slip increases with temperature. Thus, it appears likely that the hypothesis of irradiation growth resulting from vacancy and interstitial clustering on preferential planes is correct.

By 1958, it was not yet clear whether the irradiation-induced dimensional change in polycrystalline material was a result of irradiation growth due to induced lattice defects, or whether it is swelling induced due to fission gases. In fact, in the 1950s, irradiation growth and swelling were not understood as separate mechanisms, and the observed changes in α-U were simply thought of as irradiation-induced dimensional changes. Later it was found that the swelling was responsible for majority of the dimensional change. At the time, there was a hypothesis that dimensional changes resulting from lattice defects would anneal out, as well. Kittel and Paine [35] suggested that the irradiation-induced dimensional changes under 573 K were not due to swelling induced by fission gases, and further macroscale studies were carried out at different irradiation temperatures and burnups to elucidate the answer. A first meta-analysis on irradiation-induced swelling above 673 K was reported by Pugh [44] in 1961. Pugh plotted the irradiation swelling versus burnup and observed significant spread in the data. It was presumed that this spread was due to differences in the sample geometry, irradiation conditions, and potentially other factors. Certain samples exhibited grain boundary cracking or catastrophic “breakaway” swelling. A breakthrough was made on the effect of irradiation temperature and α-U swelling when the data from Pugh’s analysis were re-analyzed by Granata and Saraceno [45]. This analysis included the effect of irradiation temperature, the data exhibited a peak in swelling at intermediate temperatures. The focus then shifted towards capturing the dependence of swelling on irradiation temperature, which was previously only studied with respect to the burnup. This new understanding of the effect of temperature on swelling lead to additional careful microstructural investigations of irradiation effects on α-U.

**Figure 7:**
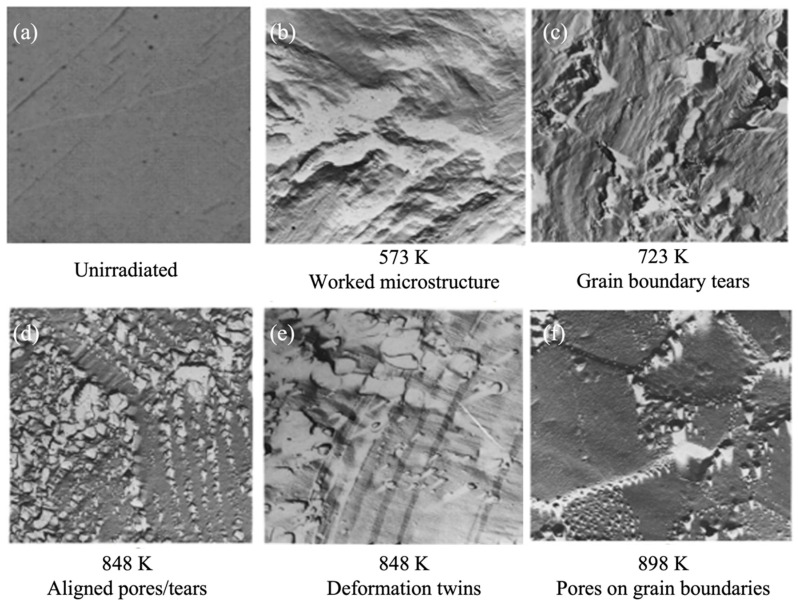
Scanning electron microscopy images of irradiated α-U showing the microstructure evolution at different irradiation temperatures [46].

**Figure 8:**
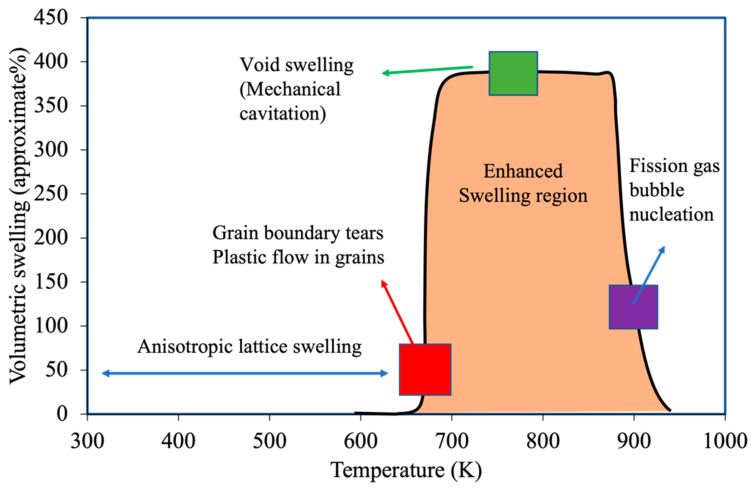
Variation in the irradiation swelling behavior and possible mechanisms at different temperature regions.

The most comprehensive and systematic study on the effect of irradiation and temperature on α-U microstructure evolution was performed by Leggett et al. [46]. In this work, irradiations of high-purity polycrystalline α-U (<150 ppm of impurity by weight) were performed over the range of 573–923 K and over a burnup range of 0.03–0.4 at% at a fast neutron flux up to 2 × 10^13^ n/cm^2^. Qualitatively different microstructures were observed depending on the irradiation temperature, potentially indicating that several different mechanisms contribute to irradiation damage in the material. The observed microscopic images at different irradiation temperatures are shown in Figure 7 compared against the unirradiated microstructure (Figure 7a). Below 623 K, a severely worked microstructure indicating plastic flow in the material was observed (Figure 7b), though no major volume change was measured. Recent modeling indicates that stresses high enough to cause plastic deformation or void formation can result in polycrystalline α-U with extremely low levels of burnup [47]. In the range of 673–773 K (Figure 7c), a similar worked microstructure was again observed, but jagged voids on the order of 1 micron also appeared, apparently the result of grain boundary tearing. Recent modeling demonstrates that this is likely due to the weaking of grain boundary at high temperatures [13]. In the range of 773–873 K (Figure 7d,e), they observed a few deformation twins and crystallographically aligned pores on the order of few microns in size. However, the grain boundaries were essentially free of pores. Above 873 K, tiny spherical pores (again in the order of 1 micron) were observed throughout the material and more dominantly at the grain boundaries (Figure 7f). Figure 8 summarizes the complex irradiation swelling behaviors as a function of temperature.

**Figure 9:**
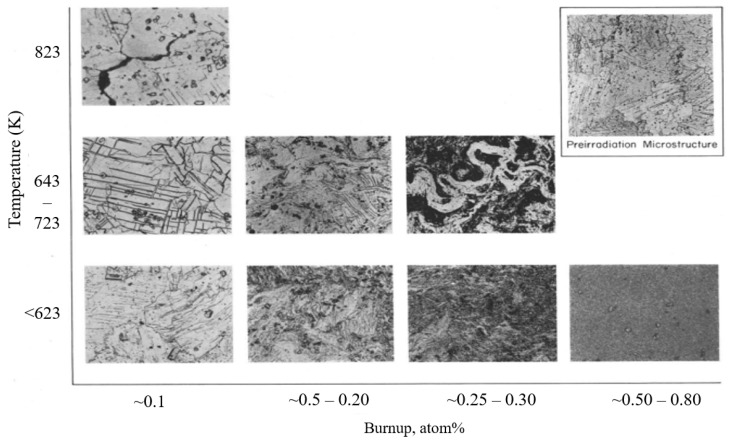
Microstructure evolution at different irradiation temperature and burnup (Reprinted/adapted with permission from Ref. [48], 2022, Elsevier).

Around the same time, Angerman and Caskey [48] reported microstructural changes in α-U over the range of 403–823 K and at burnups of 0.05 and 0.77 at%. The reactor conditions and neutron flux were not provided. Overall, their observations were very similar to that of Leggett et al. though this study focused on a somewhat lower temperature range and the samples in this study were of lower purity (~1000 ppm impurities by weight). Like Leggett et al., Angerman and Casky also observed several twin systems in most grains and intersecting twins were observed in some areas. Slip within the grains also took place, as evidenced by the bending of the twins. At higher burnups, more distorted grain structures were observed, likely due to deformation by slip. Small fission gas bubbles were uniformly distributed in the samples irradiated below 673 K, similar to other observations [44]. The authors observed the formation of irregular cavities in several samples irradiated above 723 K, which contributed significantly to the overall swelling. The metallographic analysis shown in Figure 9 reveals large cavities with a range of length (10–200 microns), especially at high irradiation temperature. The cavities were mostly found in grain boundaries or junctions with angular incisions. Angerman and Caskey [48] reported pronounced variations in microstructure and cavitation (formation of mechanical cavities/voids) with burnup, as shown in Figure 9. At burnups below 0.1 at%, the grain structure of the uranium was easily recognizable regardless of the irradiation temperature, though some grain boundary cracking was observed at high irradiation temperatures. This grain boundary cracking is likely a result of thermal expansion stress at high temperature, hypothesized in [49]. Thermal cycling experiments without irradiation found that polycrystalline samples deformed significantly and developed interior cracks with about 100 K temperature difference, and recent modeling has shown that thermal stresses arising from the negative thermal expansion coefficient is sufficient to cause cracking after a temperature change of about 100 K in irradiated α-U. At burnups of 0.15 to 0.3 at%, the grain structure became twisted and distorted as the result of deformation by slip. Uniform and fine “marbleized” structures were observed at burnups of 0.6 to 0.8 at%, though data are only available only at temperatures below 623 K.

The formation mechanisms of the multiple morphologies of irradiation-induced swelling within α-U (jagged cavities along grain boundaries, small aligned or non-aligned pores, massive cavities) is still an area of active research. Historically, the research community was unsure whether small pores (round spaces observed in the matrix) (observed in [46]) were voids or fission gas bubbles; they were later confirmed to be voids [48]. This uncertainty originated from the fact that no advanced electron microscopy was used in these investigations, generally because they were still new tools or not even invented yet. Fission bubble growth was argued to be negligible at the burnups for which these pores were observed [44], though a few arguments were put forward to support the assertion that the observed pores were gas bubbles which then grew enormously, leading to high swelling above 723 K. Pugh [44] suggested that the large “breakaway swelling” volumes were formed by the diffusion of gaseous fission products to alpha grain boundary cracks, ultimately driving plastic deformation of the matrix. Speight and Greenwood [50] later proposed that dislocation sweeping could promote the breakaway condition by increasing the average bubble size through bubble coalescence. It was also demonstrated that enhanced swelling may arise in α-U for >823 K if the intergranular stresses due to irradiation growth are high enough to move dislocations. Conversely, later studies [51] show clear evidence that certain porosity is due to mechanical cavitation and void formation. Recent modeling work has indicated that mechanical tearing along grain boundaries can arise from thermal stresses and irradiation growth stresses [13,47,49], but the nucleation mechanism of intergranular voids remains unclear. Twin boundaries were implicated in the formation of crystallographically aligned voids [46], though later work hypothesized they are void superlattices. Fission gases and vacancy agglomeration are both considered as mechanisms for void formation in α-U. To validate any of these hypotheses, the atomistic behavior of collision cascades and point defects in α-U and their collective behavior at diffusional length and time scales at low burnups must be studied with advanced characterization and simulation techniques. In addition, although several experimental investigations indicate that impurities affect the irradiation growth and swelling rate [33,51], there remains no comprehensive analysis of the role of these impurities in controlling irradiation growth and swelling behavior.

### 3.2. Irradiation Effect on Mechanical Properties

Although several attempts have been made to understand the effect of irradiation on mechanical properties of α-U, such as ductility, hardness and creep, the results of these studies have been contradictory. A common observation, however, is the increased hardness of α-U, which was hypothesized to be a result of fission product impurity, irradiation-induced voids or dislocation loop formation. In 1958, Kittel and Paine [35] tested polycrystalline natural uranium in the Materials Testing Reactor and found that the hardness increased with burnup, and that annealing did not restore the hardness. Similar observations were also made by Loomis et al. [42] on single crystalline α-U in 1964. However, a saturation in hardness was observed at a fluence of approximately 1 × 10^17^ neutrons/cm^2^. Both studies irradiated material at relatively low temperatures of up to 493 K and neither study provided information about the purity of the samples. Although radiation hardening is well known to occur due to the impediment of dislocation motion by defect loops, the reason for the observed yield strength saturation is unclear.

**Figure 10:**
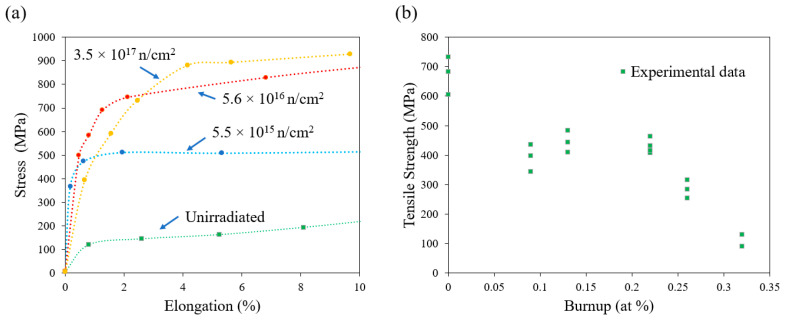
Irradiation effect on mechanical properties of (**a**) single crystal subjected to different irradiation fluences (Reprinted/adapted with permission from Ref. [52], 2022, Springer Nature) and (**b**) polycrystalline α-U (Reprinted/adapted with permission from Ref. [52], 2022, Springer Nature).

The effect of irradiation on the creep and ductility of single crystal α-U remains unclear. High-purity single crystals of α-U were irradiated and then mechanically tested, and the single crystals retained considerable ductility (Figure 10a). Conversely, significantly lowered elongations were observed in polycrystalline α-U by Vorob’ev [52] and Konobeevskii et al. [53] (Figure 10b). Konobeevskii et al. [53] attributed such reduction to the formation of numerous internal cracks in polycrystalline metal as a result of irradiation. The initial study on creep testing in irradiated U was also reported by Konobeevsky et al. [53], who irradiated the material and then creep-tested it out of pile. The creep rate was increased by a factor of 1.5 to 2 versus the un-irradiated material. Following this, Roberts and Cottrell [54] conducted in situ creep testing in an irradiation environment, and observed a similar enhancement in creep during irradiation.

Although the mechanical behavior of irradiated α-U varies significantly within the literature, it appears that while single crystal material retains its ductility, the observed loss of strength and ductility in polycrystalline material is likely the result of the presence of grain boundaries. In fact, the possibility of superplasticity in irradiated polycrystalline α-U has been discussed in [55,56]. Though internal stress-driven plasticity (a phenomenon also found to be responsible for generating environmental superplasticity in other materials [57]) is proposed to contribute to the observations in [46], there is a need for more comprehensive mesoscale modeling of irradiation considering the effects of plasticity and grain-boundary interactions. Internal stress-driven plasticity under irradiation could arise from the anisotropic radiation growth of differently oriented grains, causing stresses high enough to initiate plastic flow. Further irradiation would continue to drive irradiation growth and stresses. Microcracks at grain boundaries could arise due to the presence of hard inclusions, insufficient slip and twinning processes to accommodate deformation across grain boundaries, or low grain boundary adhesion.

However, detailed comparisons of mechanical behavior in-pile and out-of-pile from the existing literature may not be meaningful, given the very different samples, irradiation and testing conditions. Many historical tests did not characterize the details in the microstructure or composition of the materials. Many of the tests on irradiated material were performed before the development of irradiation damage models (Kinchin–Pease, Norgett—Robinson–Torrens) that calculate displacements per atom (dpa), and thus neutron fluxes, fluences, and spectra need to be reported to obtain an accurate understanding of the amount of irradiation damage. Furthermore, the irradiation temperatures were historically not well controlled or measured. Finally, given the difference in the purity level, grain size and fabrication methods, all of which could affect the irradiation behavior and mechanical response, more detailed testing is needed.

### 3.3. Irradiation Effects on Thermal and Electrical Properties

Neutron irradiation increases the electrical resistivity and thermal conductivity of α-U, but the specific relationship is not well understood. A direct correlation was observed between the irradiation-increased resistivity and single crystal irradiation growth in all the available studies, albeit with limitations on the burnup range investigated. The effect of thermal neutron irradiation on the electrical resistivity of lineage single crystals (<600 ppm impurities) was studied by [42] in the CP-5 reactor up to 550 K. For a constant irradiation temperature, electrical resistivity increased with irradiation dose and was also proportional to the increase in irradiation growth strain. However, the increase in resistivity with irradiation dose is reported to saturate [42], contrary to the dimensional changes induced by irradiation growth.

Though the very few results about the effects of irradiation on the thermal properties of α-U are non-conclusive given the uncontrolled testing performed in 1960s, the existing results and estimates do reveal that the thermal conductivity of irradiated α-U is impacted. The overall dependence is governed by various phenomena including lattice resistance imposed by defects, microstructural damage on the grain boundaries, porosity development and the presence of fission gases. Brailsford and Major [58] theoretically estimated the reduction in thermal conductivity as well as electrical resistivity due to irradiation in the form of an equation (Equation (1)), considering various key factors,

(1) $$R=150\;\frac{\left(D_{0}-D_{1}\right)}{D_{0}}+3\left(2b\right)+0.3$$

where *R* is the % reduction in the thermal conductivity, *D*_0_ and *D*_1_ are the initial and final density (g/cm^3^) (evaluated through any applicable swelling model) and b is burnup (at %). The burnup dependence implicitly accounts for changes due to lattice strains, lattice defects and fission gas bubbles and voids. Though the estimate considers several factors, several simplifying assumptions were made. Although the analysis considers the increased resistivity from irradiation effects, the effect of dislocation loops were totally neglected [59]. However, the effect of dislocation loop dynamics is expected to play a vital role in the irradiation growth rate. Given the strong, suggestive results of the dependence of electrical resistivity on the irradiation growth rate and similar mechanisms responsible for thermal resistivity, more comprehensive models need to be developed.

To date, there is no reliable experimental analysis to validate Equation (1). However, recent DFT studies by Peng et al. [60] estimate the effect of U vacancies, U interstitials and Zr substitution on the thermal conductivity. Although Equation (1) does not include temperature dependence, there is reasonable order-of-magnitude agreement between its predictions and the DFT studies. Among the three types of defects studied by DFT, U vacancies have the most substantial impact on thermal conductivity. Similar to the case of irradiation effects on dimensional changes and mechanical properties, further experimental investigations are required to understand the effect of irradiation on α-U’s thermal conductivity and electrical resistivity under carefully controlled conditions, including specification of the initial purity, the metallurgical condition of the specimen and the details of the irradiation (temperature, flux, fluence and neutron spectrum). Simultaneous measurements of the electrical resistivity and thermal conductivity during irradiation would be valuable to test the validity of the Wiedemann–Franz law for irradiated specimens, which forms the basis of Brailsford and Major’s theoretical model.

The effect of irradiation on the superconductivity of α-U is a fundamental area of interest that could receive more attention. Existing results suggest that irradiation could affect the superconductivity of materials in two different ways: First, extended defects, such as column-shaped amorphous regions, act as strong, correlated pinning centers and usually increase the critical current density substantially. Second, point defects influence the microscopic parameters responsible for superconductivity, thus affecting the critical temperature and the critical fields. Analysis of the irradiation effects could provide a strong fundamental basis to characterize the intrinsic superconductivity of uranium as well as quantify the impact of irradiation-induced defects.

## 4. Fundamental Research Needs

The objective of this review is to consolidate and synthesize research efforts to date to understand the irradiation damage and effects in α-uranium. This review surveys basic physical properties of α-U and the effects of irradiation damage, including microstructural changes, dimensional changes, mechanical properties and physical properties. The anomalous and complex behaviors of α-U are particularly highlighted, and further research in these topics will provide new insights into the irradiation damage mechanisms of α-U. With advanced characterization and modeling techniques, several important research gaps can be investigated.

Irradiation conditions (such as temperature and burnup), microstructure (such as grain structures and porosity), chemical composition (such as minor elements and impurities) and internal stress have profound effects on the irradiation response of α-U. However, the understanding is generally qualitative in nature, rather than quantitative, and several mechanisms driving irradiation response still require explanation. Both experiments and modeling will be needed to answer these questions, and special care needs be taken to characterize the initial conditions of the material to determine quantitative behaviors.

In single-crystal α-U, the temperature-dependent irradiation growth behavior remains to be validated (reproduced with new experiments) and explained. Fundamentally, the mechanism of irradiation growth remains to be determined, and requires both advanced characterization (e.g., transmission electron microscopy and positron annihilation spectroscopy) and atomistic modeling and simulation (point defect migration and clustering behavior) to fully answer. Two other major questions are why the irradiation growth rate drastically increases at cryogenic temperatures and whether irradiation growth stops at temperatures near the β phase boundary. The irradiation response of polycrystalline α-U is driven by the interplay of single-crystal behaviors and the presence of grain boundaries. Recent modeling has indicated that grain boundary adhesion decreases at higher temperatures and that irradiation growth in polycrystalline material can cause stresses up to hundreds of megapascals, sufficient to drive plastic flow or cracking. However, the migration of point defects under stress fields and their interaction with sinks such as grain boundaries, voids and second phase precipitates remains to be studied.

The effect of chemistry/impurities on the irradiation behavior is not well understood. Most of the available data are from experiments performed on samples with different impurity levels. As mentioned earlier, impurities have a profound impact on irradiation growth and swelling behavior. Certain impurities are known to reduce irradiation growth and swelling due to precipitate formation, solute-defect interactions or other such mechanisms. In addition, the impurities were also known to affect the nucleation of bubbles and cavities, leading to significant variations in mechanical behaviors. Highly pure α-U specimens are recommended for benchmark irradiation studies.

The role of internal stresses on sample auto-deformation or during mechanical testing needs to be further studied. Due to the significant thermal expansion anisotropy, bulk polycrystalline α-U is likely always experiencing microstructural stresses, except after proper heat treatments. A temperature change of 50–100 K is sufficient to induce stresses on the order of hundreds of megapascals, large enough to drive plastic flow or cracking. Irradiation growth-induced stresses can also cause internal stresses on the order of hundreds of megapascals with very low levels of burnup. As a result, internal stress could be an important mechanism driving plasticity or superplasticity in α-U. 

## Data Availability

The data are available on request from the corresponding author.

## References

[1] Hofman G., Walters L., Bauer T. (1997). Metallic fast reactor fuels. Prog. Nucl. Energy.

[2] Varaine F., Stauff N., Masson M., Pelletier M., Mignot G., Rimpault G., Zaetta A., Rouault J. Comparative review on different fuels for GEN IV Sodium Fast Reactors: Merits and drawbacks. Proceedings of the International Conference on Fast Reactors and Related Fuel Cycles (FR09).

[3] King P.L. (2021). Advanced Fuels Campaign Execution Plan.

[4] Bush S. (1958). Irradiation Effects in Uranium. Fuel Elements Conference: Paris, France, 18–23 November 1957. Sessions IV, V, VI, and VII.

[5] Lander G.H., Fisher E.S., Bader S.D. (1994). The solid-state properties of uranium A historical perspective and review. Adv. Phys..

[6] Barrett C.S., Mueller M.H., Hitterman R.L. (1963). Crystasl Structure Variations in Alpha Uranium at Low Temperatures. Phys. Rev. (Ser. I).

[7] Beeler B., Deo C., Baskes M., Okuniewski M. (2013). First principles calculations of the structure and elastic constants of α, β and γ uranium. J. Nucl. Mater..

[8] Huang -Y.G., Wirth B.D. (2011). First-principles study of diffusion of interstitial and vacancy in α U–Zr. J. Phys. Condens. Matter.

[9] Beeler B., Zhang Y., Okuniewski M., Deo C. (2018). Calculation of the displacement energy of [alpha] and [gamma] uranium. J. Nucl. Mater..

[10] Beeler B., Mahbuba K., Wang Y., Jokisaari A. (2021). Determination of Thermal Expansion, Defect Formation Energy, and Defect-Induced Strain of α-U Via ab Initio Molecular Dynamics. Front. Mater..

[11] Vitova T., Pidchenko I., Fellhauer D., Bagus P.S., Joly Y., Pruessmann T., Bahl S., González-Robles E., Rothe J., Altmaier M. (2017). The role of the 5f valence orbitals of early actinides in chemical bonding. Nat. Commun..

[12] Booth C., Jiang Y., Wang D., Mitchell J., Tobash P., Bauer E., Wall M., Allen P., Sokaras D., Nordlund D. (2012). Multiconfigurational nature of 5f orbitals in uranium and plutonium intermetallics. Proc. Natl. Acad. Sci. USA.

[13] Mahbuba K., Beeler B., Jokisaari A. (2021). Evaluation of the anisotropic grain boundaries and surfaces of α-U via molecular dynamics. J. Nucl. Mater..

[14] Mott N.F. (1946). German Physical Society in the British Zone. Nature.

[15] Matthias B.T., Geballe T.H., Corenzwit E., Andres K., Hull G.W., Ho J.C., Phillips N.E., Wohlleben D.K. (1966). Superconductivity of Beta-Uranium. Science.

[16] Geballe T.H., Matthias B.T., Andres K., Fisher E.S., Smith T.F., Zachariasen W.H. (1966). Superconductivity of Alpha-Uranium and the Role of 5 f Electrons. Science.

[17] Steinitz M.O., Burleson C.E., Marcus J.A. (1970). Low-Temperature Phase Transitions in Alpha Uranium. J. Appl. Phys..

[18] Lander G. (1982). Charge-density waves in alpha-uranium: A story of endless surprises. J. Magn. Magn. Mater..

[19] Smith T., Fisher E. (1973). Superconductivity and phase transitions in single-crystal and polycrystal α-u at high pressure. J. Low Temp. Phys..

[20] Van Gennep D. (2012). Charge Density Waves and Superconductivity in Alpha-Uranium. Honors Thesis.

[21] Arajs S., Flora R.H., Anderson E.E. (1970). Electrical resistivity and thermoelectric power of polycrystalline uranium at elevated temperatures. J. Nucl. Mater..

[22] Hin C. (2018). Thermal Conductivity of Metallic Uranium.

[23] Fisher E. (1966). Temperature dependence of the elastic moduli in alpha uranium single crystals, part iv (298° to 923° K). J. Nucl. Mater..

[24] Gubel N.R., Eckelmeyer K.H., Johnson K.N., Jackson M.J., Morrell J.S. (2013). Introduction to Uranium, in Uranium Processing and Properties.

[25] Franz R., Wiedemann G. (1853). On the thermal conductivity of metals. Ann. Phys..

[26] Bridge J.R., Schwartz C.M., Vaughan D.A. (1956). X-ray Diffraction Determination of The Coefficients of Expansion of Alpha Uranium. JOM.

[27] Lloyd L.T., Barrett C.S. (1966). Thermal expansion of alpha uranium. J. Nucl. Mater.

[28] Ren Z., Wu J., Ma R., Hu G., Luo C. (2016). Thermodynamic properties of α-uranium. J. Nucl. Mater..

[29] Lehr P., Langeron J. (1957). Dilatometric study of uranium in the alpha phase. Rev. Met..

[30] Flotow H.E., Lohr H.R. (1960). The heat capacity and thermodynamic functions of uranium from 5 to 350° K. J. Phys. Chem..

[31] Flotow H.E., Osborne D.W. (1966). Heat Capacity of Alpha Uranium from 1.7 to 25 °K. Phys. Rev. (Ser. I).

[32] Loomis B., Gerber S. (1965). The effect of pre-irradiation heat treatment and a brief postirradiation beta anneal on swelling of alpha uranium. J. Nucl. Mater..

[33] Harrison J. (1967). The growth of gas bubbles in a stressed medium and the application to stress enhanced swelling in alpha uranium. J. Nucl. Mater..

[34] Pugh S. (1952). The Growth of Alpha-Uranium under Irradiation.

[35] Kittel J.H., Paine S.H. (1958). Effect of Irradiation on Fuel Materials.

[36] Paine S., Kittel J. (1958). Preliminary Analysis of Fission-Induced Dimensional Changes in Single Crystals of Uranium.

[37] Cahn R. (1953). Plastic deformation of alpha-uranium; twinning and slip. Acta Met..

[38] Loomis B.A., Blewitt T.H., Klank A.C., Gerber S.B. (1964). Elongation of uranium single crystals during neutron irradiation. Appl. Phys. Lett..

[39] Seigle L.L., Opinsky A.J. (1957). Mechanism of Dimensional Instability of Uranium. Nucl. Sci. Eng..

[40] Buckley S. Properties of Reactor Materials and the Effects of Radiation Damage. Proceedings of the International Conference held at Berkeley Castle.

[41] Hudson B., Westmacott K.H., Makin M.J. (1962). Dislocation loops and irradiation growth in alpha uranium. Philos. Mag..

[42] Loomis B.A., Gerber S.B. (1968). Length and electrical resistivity changes of neutron irradiated uranium. Philos. Mag..

[43] Choi S.I., Kim J.H. (2013). Radiation-induced dislocation and growth behavior of zirconium and zirconium alloys—A review. Nucl. Eng. Technol..

[44] Pugh S. (1964). Swelling in alpha-uranium due to irradiation in the range 400° to 650 °C. J. Nucl. Mater..

[45] Granata S., Saraceno F. (1963). The relationship between burn-up, temperature, and swelling in alpha uranium. J. Nucl. Mater..

[46] Leggett R.D., Mastel B., Bierlein T.K. (1964). Irradiation Behavior of High-Purity Uranium.

[47] Jokisaari A. (2020). Irradiation-induced internal stresses in polycrystalline α-uranium: A mesoscale mechanical approach. Comput. Mater. Sci..

[48] Angerman C., Caskey G. (1964). Swelling of uranium by mechanical cavitation. J. Nucl. Mater..

[49] Rezwan A.A., Jokisaari A.M., Tonks M.R. (2021). Modeling brittle fracture due to anisotropic thermal expansion in polycrystalline materials. Comput. Mater. Sci..

[50] Speight M., Greenwood G. (1965). The effects of dislocation movement in enhancing swelling in α-uranium during irradiation. J. Nucl. Mater..

[51] McDonell W.R. (1972). Irradiation Swelling and Growth in Uranium and Other Anistropic Metals. Trans. Am. Nucl. Soc..

[52] Vorob’Ev M.A., Golovchenko Y.M., Davydenko A.S., Bychkov B.A. (1970). Mechanical properties of irradiated uranium. Sov. At. Energy.

[53] Konobeevskii S.T., Pravdyuk N.F., Dubrovin K.P., Levitskii B.M., Panteleev L.D., Golianov V.M. Structural properties of irradiation Uranium. Proceedings of the United Nations International Conference on the Peaceful Uses of Atomic Energy.

[54] Roberts A.C., Cottrell A.H. (1956). LXXII. Creep of alpha uranium during irradiation with neutrons. Philos. Mag..

[55] Sherby O.D., Caligiuri R.D., Kayali E.S., White R.A. (1981). Fundamentals of Superplasticity and Its Application. Advances in Metal Processing.

[56] Johnson R., Sykes E. (1966). Enhancement of Ductility in α-Uranium. Nature.

[57] Padmanabhan K.A., Davies G.J. (1980). Superplasticity.

[58] Brailsford A. (1963). The resistivity of interstitial atoms and vacancies in α-uranium. J. Nucl. Mater..

[59] Konobeevski S. (1964). Effect of neutron irradiation on the electrical resistivity of uranium. J. Nucl. Mater..

[60] Peng J., Deskins W.R., Malakkal L., El-Azab A. (2021). Thermal conductivity of *α*-U with point defects. J. Appl. Phys..

